# Effect of TAVR Approach and Other Baseline Factors on the Incidence of Acute Kidney Injury: A Systematic Review and Meta-Analysis

**DOI:** 10.1155/2022/3380605

**Published:** 2022-10-27

**Authors:** Hossam Alzu'bi, Anan Abu Rmilah, Ikram-UI Haq, Babikir Kheiri, Ahmad Al-abdouh, Bashar Hasan, Omar Elsekaily, Suhaib Jaber, Ibraheem Qaisi, Asil Yagmour, Hamada Dajani, Azza Ahmed, Ahmed elhag, Kianoush Kashani, Abhishek Deshmukh

**Affiliations:** ^1^Department of Cardiovascular Medicine, Mayo Clinic, Rochester, MN, USA; ^2^Department of Internal Medicine, Mayo Clinic, Rochester, MN, USA; ^3^Knight Cardiovascular Institute, Oregon Health & Science University, Portland, OR, USA; ^4^Department of Medicine, Ascension Saint Agnes Hospital, Baltimore, MD, USA; ^5^Evidence-Based Practice Research Program, Mayo Clinic, Rochester, MN, USA; ^6^Department of Internal Medicine, Dr. Sulaiman Al Habib Hospital, Riyadh, Saudi Arabia; ^7^An-Najah National University School of Medicine, Nablus, State of Palestine; ^8^Al Quds University School of Medicine, Hebron, State of Palestine; ^9^Division of Nephrology and Hypertension, Mayo Clinic, Rochester, MN, USA

## Abstract

**Background:**

Acute kidney injury (AKI) is a well-known complication following a transcatheter aortic valve replacement (TAVR) and is associated with higher morbidity and mortality.

**Objective:**

We aim to compare the risk of developing AKI after transfemoral (TF), transapical (TA), and transaortic (TAo) approaches following TAVR.

**Methods:**

We searched Medline and EMBASE databases from January 2009 to January 2021. We included studies that evaluated the risk of AKI based on different TAVR approaches. After extracting each study's data, we calculated the risk ratio and 95% confidence intervals using RevMan software 5.4. Publication bias was assessed by the forest plot.

**Results:**

Thirty-six (36) studies, consisting of 70,406 patients undergoing TAVR were included. Thirty-five studies compared TF to TA, and only seven investigations compared TF to TAo. AKI was documented in 4,857 out of 50,395 (9.6%) patients that underwent TF TAVR compared to 3,155 out of 19,721 (16%) patients who underwent TA-TAVR, with a risk ratio of 0.49 (95% CI, 0.36–0.66; p < 0.00001). Likewise, 273 patients developed AKI out of the 1,840 patients (14.8%) that underwent TF-TAVR in contrast to 67 patients out of the 421 patients (15.9%) that underwent TAo-TAVR, with a risk ratio of 0.51 (95% CI, 0.27–0.98; *p* = 0.04). There was no significant risk when we compared TA to TAo approaches, with a risk ratio of 0.89 (95% CI, 0.29–2.75; *p* = 0.84).

**Conclusion:**

The risk of post-TAVR AKI is significantly lower in patients who underwent TF-TAVR than those who underwent TA-TAVR or TAo-TAVR.

## 1. Introduction

Aortic stenosis (AS) is the most prevalent valvular heart disease in developed countries, estimated to be 2.5% of the US population [1]. It is more common in older adults >65 years old, predominately secondary to progressive degenerative changes [1]. Surgical aortic valve replacement (SAVR) has been the standard treatment for patients with severe symptomatic AS. Over the last decade, transcatheter aortic valve implantation (TAVR) has emerged as a promising therapy for most patients with severe symptomatic AS and is recommended for inoperable or moderate to high-risk surgery patients, according to the American College of Cardiology/American Heart Association Joint Committee [2]. Multiple approaches for performing TAVR have been developed over the last decade with the transfemoral (TF) approach being the most prevalent. Alternative approaches include transapical (TA) and transaortic (TAo) [3].

Complications related to TAVR are well described in the literature, especially the need for pacemaker implantation, acute kidney injury (AKI), and atrial fibrillation (AF). AKI, defined as a rapid increase of serum creatinine, decrease in urine output, or both, is a common complication following TAVR estimated to be 22% [4]. Patients who develop AKI post-TAVR have poor in-hospital outcomes, and higher in-hospital and late mortality rates [5, 6]. AKI after TAVR was one of the “big 5” complications identified in the landmark PARTNER trials [7–9]. Multiple meta-analyses have confirmed that TAVR is associated with a significantly lower AKI risk than SAVR [10–14]. Still, no recent meta-analysis in the last three years evaluated the risk of developing AKI post-TAVR based on differences in TAVR approaches.

Previous meta-analyses showed considerable evidence in different TAVR access can lead to different outcomes; however, the number of included studies was small [15, 16]. Our study represents the largest, most updated meta-analysis. Therefore, we aimed to investigate AKI incidence in patients undergoing TAVR using different approaches.

## 2. Methods

We completed this systematic review in accordance with PRISMA standards for systematic review and meta-analysis quality reporting.

### 2.1. Search Strategy

Medline and EMBASE databases were searched on February 2021 for all relevant studies published from January 2009 through January 2021 by a professional librarian. Comparative observational studies which assessed the risk of developing AKI in TF, TA, and TAo TAVR approaches in adult patients were included. The following search terms were used: (aortic stenosis or AS), (transcatheter aortic valve implantation or TAVR), (transcatheter aortic valve implantation or TAVI), (transfemoral or TF), (transapical or TA), (transaortic or TAo), (approach), and (acute kidney injury or AKI).

### 2.2. Inclusion Criteria

Inclusion criteria were as follows: studies must be either observational (cohort, case-control) or randomized controlled trials (RCTs) comparing the risk of developing AKI among different TAVR approaches (TF, TA, and TAo) for patients with severe symptomatic aortic stenosis.

### 2.3. Exclusion Criteria

Our exclusion criteria included the following: nonoriginal studies (commentaries, editorials, reviews, and meta-analysis), abstracts with insufficient data, animal studies, pediatric and pregnant patients' populations, and studies that did not report AKI outcomes separately for TF-, TA-, and TAo-TAVR approaches. In studies with overlapping patient populations, the study with a larger sample size was included.

### 2.4. Study Selection

Study selection was completed independently and in duplicate by two trained reviewers (AA and AY) and coordinated using Covidence systematic review software (Veritas Health Innovation, Melbourne, Australia). Title and abstract screening were initially performed, followed by full-text screening to determine the final eligibility. Studies with incomplete information after author contact were excluded. Conflicts were resolved via consensus with a third reviewer (HA). A kappa statistic was calculated to assess agreement [17].

### 2.5. Data Extraction

Data related to the design of included studies, publication date, country of origin, patient recruitment period, baseline characteristics of the participants, TAVR access site, and follow-up duration were abstracted by two reviewers (BH, OE) using standardized Excel sheets as shown in (Supplemental Table 1).

### 2.6. Statistical Analysis

Categorical variables were expressed as a number of cases (*n*) and percentages (%), whereas continuous variables were represented as means and standard deviations (SD). A risk ratio (RR) with a 95% confidence interval (CI) was calculated for each study and pooled across studies using the Mantel–Haenszel method under a random-effects model. Heterogeneity was evaluated using the *I*^2^ index and Cochrane *Q* chi-squared test. Study heterogeneity was considered significant at P < 0.10 or *I*^2^ > 50%. Statistical significance was set at P < 0.05. We used the studies that compare two groups; patients who developed AKI after TAVR and those who did not to explore which baseline parameters can significantly predict post-TAVR AKI. We included the baseline variables in which datasets from 2 or more studies were available. As given in each study, we extracted the total sample size of post-TAVR AKI and non-AKI groups, and the number of patients having a specific predictor of interest in each group. Accordingly, crude RRs were directly calculated using the Mantel–Haenszel method under a random-effects model and entered into the primary analysis. A Forest plot of summary crude RRs of each assessed predictor for AKI among patients undergoing TAVR was included in our study. All statistical analyses were conducted using RevMan version 5.4.

### 2.7. Risk of Bias Assessments

The risk of bias was evaluated with the Newcastle–Ottawa Scale [18] (for observational studies) by two reviewers (BH or OE) working independently. A third reviewer (HA) resolved disagreements. Studies were classified into one of three categories: (a) low risk 6–7 points (b) moderate 3–5 points, and (c) high risk 0–2 points (Supplemental Table 2).

### 2.8. Publication Bias

**Figure 1:**
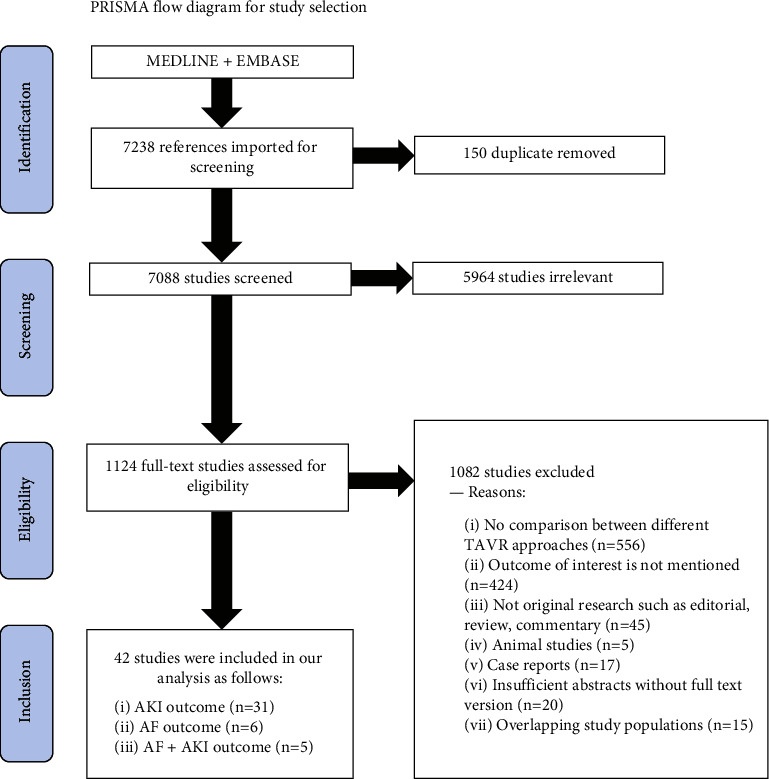
PRISMA flow chart for the systematic review and meta-analysis study.

Publication bias was assessed by visual inspection of the funnel plot for AKI outcome (Figure 1. The absence of publication bias was determined when all studies (dots) exist within the funnel plot symmetrically.

## 3. Results

**Figure 2:**
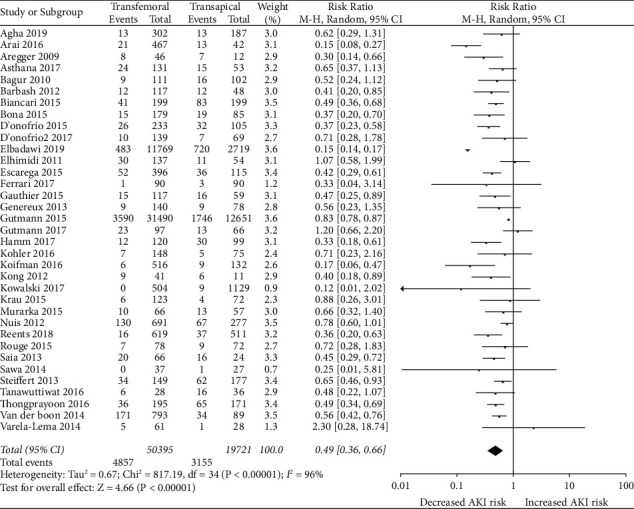
Forest plot comparing the risk of developing AKI between TF and TA TAVR approaches.

As shown in (Figure 2), 7,088 potentially relevant articles were retrieved by the initial search after removing 150 duplicate items. A total of 5,964 did not meet our inclusion criteria based on screening at the title and abstract level. As a result, 1,124 articles were reviewed for final eligibility in the full-text screening phase. We excluded 1,088 articles based on the predefined inclusion and exclusion criteria. Eventually, 36 articles with a total of 70,406 patients were included, 35 studies compared TF to TA, and only 7 investigations compared TF to TAo.

The participant's baseline characteristics, access site, and design of the included studies are shown in **(**Supplemental Table 1). The patients' mean age in the included studies ranged from 78.8 to 85.8 years, and the proportion of enrolled male patients varied from 35% to 64%. According to the Valve Academic Research Consortium (VARC) definitions of AKI were used by the vast majority of the included studies except for three studies [19–21] that did not specify their criteria for AKI diagnosis. Interestingly, sensitivity analysis before and after the exclusion of these three studies did not show any significant differences in the results for patients that underwent TF-TAVR vs. TA-TAVR [RR: 0.46 (95% CI, 0.34–0.64; p < 0.00001), compared to RR 0.49 (95% CI, 0.36–0.66; p < 0.00001)]). Therefore, we included all 36 studies in our final analysis regardless of the AKI definition.

### 3.1. Outcomes

#### 3.1.1. Acute Kidney Injury after TAVR

**Figure 3:**
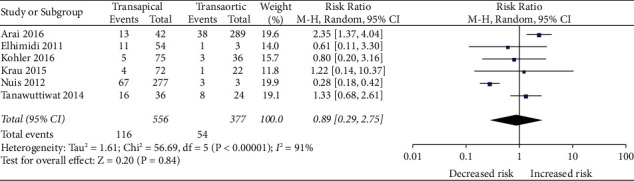
Forest plot comparing the risk of developing AKI between TF and TAo TAVR approaches.

*(1) TAVR-TF vs TAVR-TA*. AKI was documented in 4,857 out of 50,395 (9.6%) patients that underwent TF-TAVR compared to 3,155 out of 19,721 (16%) patients in TA-TAVR group, with a risk ratio of 0.49 (95% CI, 0.36–0.66; p < 0.00001) (Figure 3).

**Figure 4:**
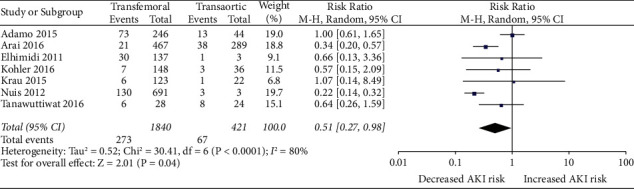
Forest plot comparing the risk of developing AKI between TA and TAo TAVR approaches.

*(2) TAVR-TF vs TAVR-TAo*. Likewise, 273 out of the 1,840 (14.8%) TF-TAVR patients developed AKI in contrast to 67 out of the 421 patients (15.9%) that underwent TAo-TAVR, with a risk ratio of 0.51 (95% CI, 0.27–0.98; *p* = 0.04) (Figure 4)

*(3) TAVR-TA vs TAVR-TAo*. A risk ratio of 0.89 (95%CI, 0.29–2.75; *p* = 0.84) was statistically insignificant in comparison between TA-TAVR and TAo-TAVR approaches (Figure 1).

*(4) Analysis of Pre-TAVR Baseline Factors*. Herein, we aimed to assess the predictability of baseline comorbidities and other factors in estimating the risk of post-TAVR AKI besides the TAVR approach. We used the studies that compare the baseline parameters between two groups; patients who developed AKI after TAVR and those who did not. We included the baseline variables in which datasets from 2 or more studies were available. Meta-analysis with forest plots for each assessed baseline factor in predicting post-TAVR AKI is shown in Supplemental Figures 1–9. In Table 1, we included a summary of crude RRs of each assessed predictor for AKI among patients undergoing TAVR. In our analysis, we found that blood transfusion (OR 2.45 95% CI 1.90–3.15, P < 0.00001), PVD (OR 1.72 95% CI 1.35–2.20, P < 0.0001), and CHF (OR 1.48 95% CI 1.10–2.00, *P* = 0.01) are independent risk factors for AKI post-TAVR.

#### 3.1.2. Mortality in Patients with Acute Kidney Injury after TAVR

**Figure 5:**
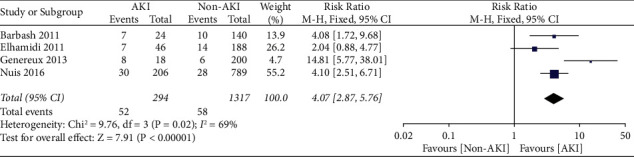
Meta-analysis of 30-day all-cause mortality in AKI versus non-AKI patients after TAVR.

*(1) 30-Day Mortality*. Our study showed that 30-day mortality was increased among patients who developed AKI after TAVR as compared to non-AKI patients. 52 out of 294 (17.7%) AKI patients and 58 out of 1317 (4.4%) non-AKI patients died within 30 days of the TAVR procedure, with a risk ratio of 4.07 (95%CI, 2.87–5.76; p < 0.00001) (Figure 5).

**Figure 6:**
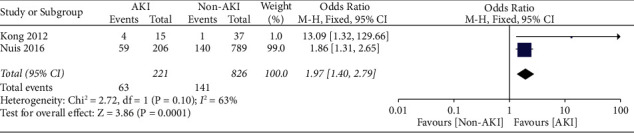
Meta-analysis of one-year all-cause mortality in AKI versus non-AKI patients after TAVR.

*(2) 1-Year Mortality*. Likewise, one-year mortality was increased among patients who developed AKI after TAVR as compared to non-AKI patients. 63 out of 221 (28.5%) AKI patients and 141 out of 826 (17.1%) non-AKI patients died within 30 days of the TAVR procedure, with a risk ratio of 1.97 (95% CI, 1.40–2.79; *p* = 0.0001) (Figure 6)

### 3.2. Bias Assessment

In accordance with the Newcastle–Ottawa Scale's scoring system, most included studies had a moderate risk of bias. A third of them were of high quality (low risk of bias). Two studies had a high risk of bias, mainly due to selection bias, inadequate comparability of cohorts' baseline prognostic factors, and lack of blinding of outcome assessors and data analysts.

**Figure 7:**
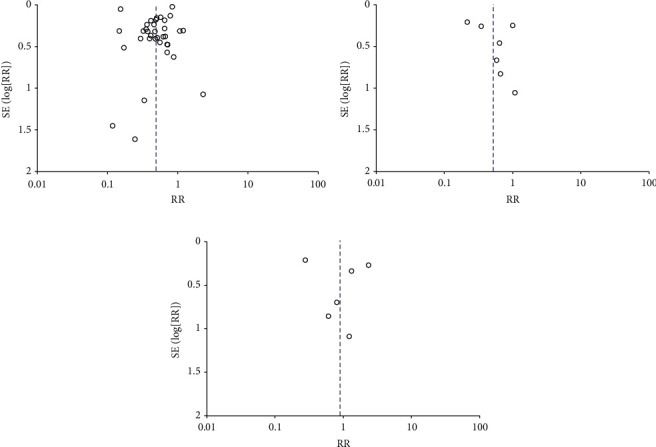
(a): Funnel plot for the risk of developing AKI between TF- and TA-TAVR approaches. (b) Funnel plot for the risk of developing AKI between TF- and TAo-TAVR approaches. (c) Funnel plot for the risk of developing AKI between TA and TAo TAVR approaches.

The meta-analysis of AKI based on TF-TAVR vs. TA-TAVR, TF-TAVR vs. TAo-TAVR approaches demonstrated symmetrically distributed studies on either side of the overall effect line (RR line) in funnel plots and therefore appear to reflect no significant publication bias in the study literature (Figure 7).

## 4. Discussion

Our meta-analysis's key finding is that the risk for developing post-TAVR AKI is significantly reduced in patients who underwent TAVR via TF access compared to those who underwent TAVR through TA or TAo accesses.

There is a disparity in the published incidence rate for developing AKI following TAVR with a range of 6 to 50% [4, 6, 19, 20]. Gargiulo G et al. 2015 meta-analysis reported that 22% of patients undergoing TAVR developed AKI [4]. In our analysis, we noticed that the composite rate of AKI within 30-days of TAVR was 8159/70817 (11.5%) with a range of 0.6–40% in individual studies. Such a wide range was attributed to the variability in several factors including baseline patient characteristics, comorbidities, the location where the study was conducted, and most importantly, the different methods used to define AKI in different studies.

The occurrence of post-TAVR AKI is associated with worse outcomes [4–6]. In our study, we found that patients who developed AKI after TAVR exhibited higher short- and long-term mortality [4–6]. Our results implied that patients who developed AKI after TAVR are at 4 times the risk of mortality at 30 days and 2 times the risk of mortality at one year compared to non-AKI patients. Gargiulo G et al. 2015 meta-analysis demonstrated a significant increase of early (23 studies; 5,563 patients; OR 5.09; 95% CI, 4.03–6.43; P < 0.00001) and one-year all-cause mortality (13 studies; 3,916 patients; OR 3.27; 95% CI, 2.42–4.42; P < 0.00001) for all patients experiencing AKI (including stages 1, 2, and 3) versus non-AKI patients [4]. Another meta-analysis published by Elhmidi et al. 2014 showed that the 30-day and 1-year mortality rates ranged from 8.8% to 44.4% and 31.5% to 55.5%, respectively [6]. They found that all included studies have shown that AKI tends to be a powerful predictor of mortality at short- and long-term follow-ups after TAVR. Such association persisted independent of baseline characteristics and periprocedural complications during TAVR [5,6]. Furthermore, the prevalence of AKI requiring renal replacement therapy RRT varies between 0% and 21%, and the discrepancy is attributed to the fact that many studies exclude patients with advanced CKD [21]. Gargiulo G et al. 2015 meta-analysis evaluated the impact of AKI after TAVR and reported that the rate of new RRT was 5.8% [4]. Elhmidi et al. 2014 revealed that approximately one-quarter of patients with post-TAVR AKI underwent RRT with a range of 2–40% in individual studies [6].

Multiple systematic reviews and meta-analyses, [10–14, 22] have confirmed that TAVR is associated with a significantly lower risk of AKI than SAVR. Still, no recent meta-analysis in the last three years evaluated the risk of developing AKI post-TAVR in different approaches to our knowledge. Thongprayoon et al. 2016 meta-analysis included seventeen studies comparing the risk of developing AKI post-TAVR between TF and TA approaches [16]. The study encompassed multiple definitions and measures of AKI, similar to our report. It included all AKI definitions in the final analysis since no significant difference was found in the sensitivity meta-analysis based on VARC-2 definitions [16]. They concluded around a 2.2-fold increase in the risk of developing AKI in the TA approach than in the TF approach [16]. Wang et al. 2017 meta-analysis examined the risk of post-TAVR AKI development according to the VARC-2 definition [23]. The study included thirteen studies and revealed that the risk of AKI is 57% less likely to occur following TF-TAVR access compared with non-TF access [23]. Ghatak et al. 2015 meta-analysis also investigated the risk of developing AKI post-TAVR according to the VARC definition, and they included nine studies [24]. They found that TF-TAVR access significantly reduced the risk of AKI by 47% compared with non-TF access [24]. Liao et al. 2017 meta-analysis included seven studies and reported the TA approach as an independent predictor of AKI post-TAVR [15]. Our research is the largest updated meta-analysis to study the association of AKI and different TAVR approaches with 70,406 patients in thirty-six studies. Our results appeared consistent with previously published meta-analyses, denoting a two-fold increase in the risk of developing AKI in patients undergoing TAVR via TA or TAo approaches compared to the TF approach. Future studies are warranted to evaluate all potential risk factors and prophylactic interventions that can mitigate the AKI risk and improve patient outcomes following TA- and TAo-TAVR approaches.

As outlined in supplemental Table 1, patients undergoing TF-TAVR had a lower prevalence of PAD than the TA and TAo patients, which might explain the lower incidence of AKI theoretically. However, some studies [15,16,25] reported TA access as an independent predictor for AKI. Other studies [15,26,27] controlled their baseline comorbidities when they compared TF to TA access using propensity matching, and TA access was still independently associated with a higher risk of AKI after TAVR. Also, non-TF access like TA and TAo are generally more invasive than TF access, leading to more hemodynamic instability and triggering the immune system to release inflammatory markers. There is a strong association between developing AKI after TAVR and higher post-procedural peaks of high-sensitivity C-reactive protein [25], which might explain the increased risk of AKI development in non-TF approaches. Cheng et al. in their 2018 meta-analysis showed the risk of blood transfusion is higher in SAVR compared to TAVR, which could explain the higher risk for post-SAVR AKI [28]. Non-TF approaches are generally at higher risk of blood transfusion which also might explain the higher risk of developing AKI [25].

### 4.1. Limitations

All our included studies were observational studies that can be more affected by confounders in contrast to randomized controlled trials due to these confounders' uneven distribution. Another limitation is that multiple methods were used by different studies to measure or define AKI. Therefore, it was not possible to perform any adjustment for baseline and procedural confounders such as contrast medium amount and baseline kidney function. However, similar results were observed on the sensitivity analysis after excluding the five studies which did not use VARC criteria.

## 5. Conclusions

The risk for post-TAVR AKI is significantly lower in patients who underwent TAVR via TF access than those who underwent TAVR through TA or TAo access. There was no significant risk difference for developing post-TAVR AKI when we compared TA to TAo approaches. Future meta-analyses based on randomized controlled trials (RCTs) are needed to reduce potential confounders between different TAVR approaches.

## Figures and Tables

**Table 1:** Table showing a summary of crude RRs of each assessed predictor for AKI among patients undergoing TAVR.

Factor	Number of studies	AKI group-total	AKI group having predictor factor	Non-AKI group-total	Non-AKI group having predictor factor	Odds ratio	Confidence interval	P value	Heterogeneity (P value, I^2^)
Male gender	8	391	187	1642	776	1.04	0.83–1.31	0.73	0.49, 0%
Smoking	2	64	4	388	34	0.79	0.09–7.23	0.84	
Hypertension	8	374	319	1642	508	2.54	0.35–18.5	0.36	< 0.0001, 92%
Diabetes mellitus	8	391	111	1642	435	1.30	0.85–2.00	0.22	0.06, 49%
Atrial fibrillation	3	248	66	1130	317	0.98	0.73–1.38	0.98	0.47, 0%
Coronary artery disease	8	391	170	1642	698	1.06	0.84–1.35	0.62	0.60, 0%
Congestive heart failure	3	246	165	1014	576	1.48	1.10–2.00	0.01	0.86, 0%
Peripheral vascular disease	7	376	138	1605	432	1.72	1.35–2.20	< 0.0001	0.90, 0%
Blood transfusion	7	376	189	1602	509	2.45	1.90–3.15	< 0.001	0.59, 0%

## Data Availability

The data supporting this study are from previously reported studies and datasets, which have been cited. The processed data are available in supplemental Table 1.

## Supplementary Materials

Supplementary Materials Supplemental Figure 1: a meta-analysis of atrial fibrillation in predicting post-TAVR AKI. Supplemental Figure 2: a meta-analysis of blood transfusion in predicting post-TAVR AKI. Supplemental Figure 3: a meta-analysis of coronary artery disease in predicting post-TAVR AKI. Supplemental Figure 4: a meta-analysis of congestive heart failure in predicting post-TAVR AKI. Supplemental Figure 5: a meta-analysis of diabetes mellitus in predicting post-TAVR AKI. Supplemental Figure 6: a meta-analysis of hypertension in predicting post-TAVR AKI. Supplemental Figure 7: a meta-analysis of male gender in predicting post-TAVR AKI. Supplemental Figure 8: a meta-analysis of peripheral vascular disease in predicting post-TAVR AKI. Supplemental Figure 9: a meta-analysis of smoking in predicting post-TAVR AKI. Table 1: risk of bias–comparative observational studies.().
