# Supplementary material for: Effect of TAVR Approach and Other Baseline Factors on the Incidence of Acute Kidney Injury: A Systematic Review and Meta-Analysis
Source: J Interv Cardiol. 2022 Oct 27;2022:3380605. doi: 10.1155/2022/3380605 (PMC9633203; doi:10.1155/2022/3380605)

Supplemental figure 1: Meta-analysis of atrial fibrillation in predicting post-TAVR AKI

	AKI +		AKI −			Odds Ratio
**Study or Subgroup**	**Events**	**Total**	**Events**	**Total**	**Weight**	**M-H, Random, 95% CI**
Barbash 2012	2	24	26	141	4.4%	0.40 [0.09, 1.82]
Genereux 2013	8	18	82	200	10.7%	1.15 [0.44, 3.04]
Nuis 2012	56	206	209	789	84.8%	1.04 [0.73, 1.46]
**Total (95% CI)**		**248**		**1130**	**100.0%**	**1.00 [0.73, 1.38]**
Total events	66		317			

Heterogeneity: $Tau^2 = 0.00$; $Chi^2 = 1.53$, df = 2 (P = 0.47); $I^2 = 0\%$

Test for overall effect: Z = 0.03 (P = 0.98)

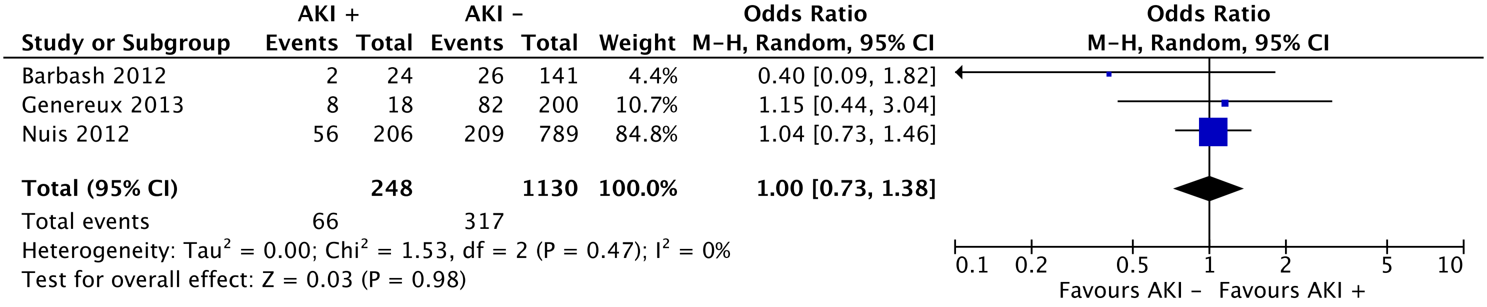

Supplemental figure 2: Meta-analysis of blood transfusion in predicting post-TAVR AKI

Study or Subgroup	AKI + Events	AKI + Total	AKI − Events	AKI − Total	Weight	Odds Ratio M–H, Random, 95% CI
Bagur 2009	19	25	85	188	6.8%	3.84 [1.47, 10.04]
Barbash 2012	15	24	55	140	7.9%	2.58 [1.05, 6.29]
Elhamidi 2011	38	46	127	188	9.3%	2.28 [1.00, 5.19]
Genereux 2013	8	18	53	200	6.5%	2.22 [0.83, 5.92]
Kong 2012	10	15	7	37	3.4%	8.57 [2.22, 33.15]
Nuis 2012	73	206	157	789	56.3%	2.21 [1.58, 3.09]
Saia 2013	26	42	25	60	9.7%	2.27 [1.02, 5.10]
**Total (95% CI)**		**376**		**1602**	**100.0%**	**2.45 [1.90, 3.15]**
Total events	189		509			

Heterogeneity: $Tau^2 = 0.00$; $Chi^2 = 4.61$, df = 6 (P = 0.59); $I^2 = 0\%$
Test for overall effect: Z = 6.99 (P < 0.00001)

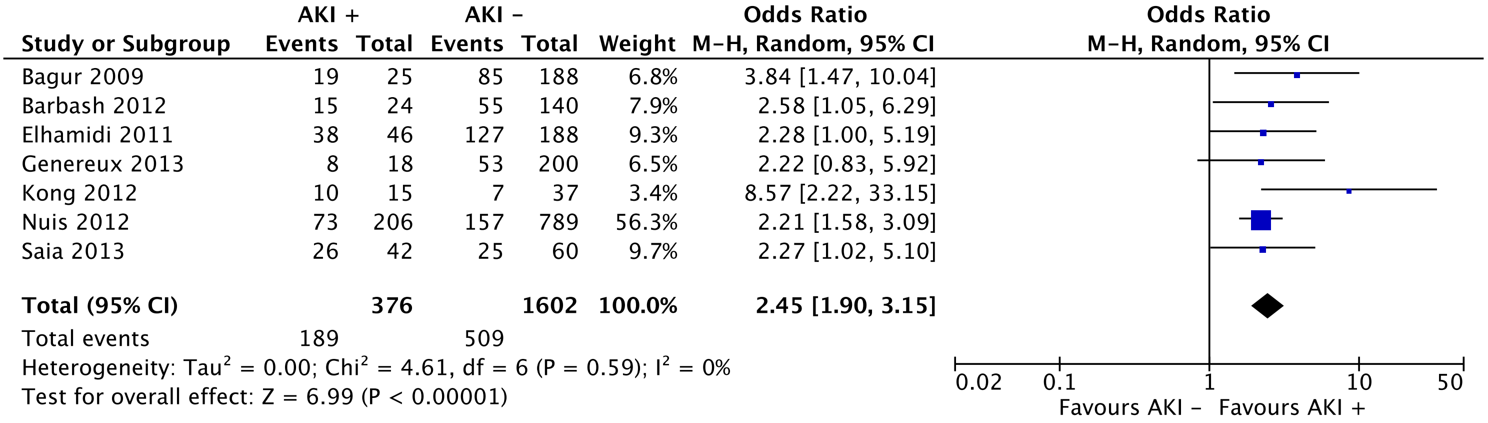

Supplemental figure 3: Meta-analysis of coronary artery disease in predicting post-TAVR AKI

	AKI +		AKI −			Odds Ratio	Odds Ratio
**Study or Subgroup**	**Events**	**Total**	**Events**	**Total**	**Weight**	**M–H, Random, 95% CI**	**M–H, Random, 95% CI**
Aregger 2009	9	15	20	39	3.9%	1.43 [0.43, 4.77]	
Bagur 2009	14	25	126	188	7.9%	0.63 [0.27, 1.46]	
Barbash 2012	15	24	77	141	7.1%	1.39 [0.57, 3.37]	
Elhamidi 2011	23	46	101	188	13.6%	0.86 [0.45, 1.64]	
Genereux 2013	9	18	80	200	6.0%	1.50 [0.57, 3.94]	
Kong 2012	14	15	26	37	1.2%	5.92 [0.69, 50.73]	
Nuis 2012	64	206	239	789	51.2%	1.04 [0.74, 1.45]	
Saia 2013	22	42	29	60	9.1%	1.18 [0.53, 2.59]	
**Total (95% CI)**		**391**		**1642**	**100.0%**	**1.06 [0.84, 1.35]**	
Total events	170		698				

Heterogeneity: $Tau^2 = 0.00$; $Chi^2 = 5.52$, df = 7 (P = 0.60); $I^2 = 0\%$

Test for overall effect: Z = 0.49 (P = 0.62)

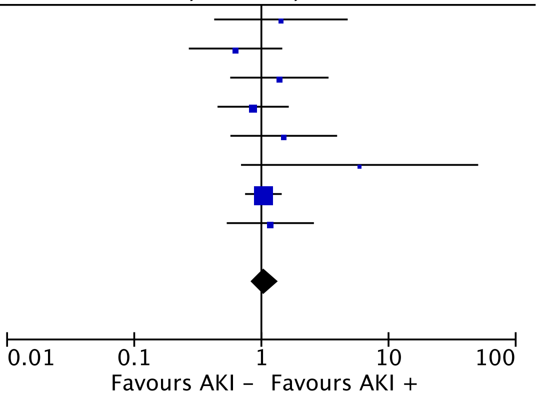

Supplemental figure 4: Meta-analysis of congestive heart failure in predicting post-TAVR AKI

Study or Subgroup	AKI + Events	AKI + Total	AKI – Events	AKI – Total	Weight	Odds Ratio M–H, Random, 95% CI
Bagur 2009	15	25	100	188	12.3%	1.32 [0.56, 3.09]
Kong 2012	15	15	34	37	1.0%	3.14 [0.15, 64.65]
Nuis 2012	135	206	442	789	86.7%	1.49 [1.08, 2.06]
**Total (95% CI)**		**246**		**1014**	**100.0%**	**1.48 [1.10, 2.00]**
Total events	165		576			

Heterogeneity: Tau^2 = 0.00; Chi^2 = 0.31, df = 2 (P = 0.86); I^2 = 0%
Test for overall effect: Z = 2.58 (P = 0.010)

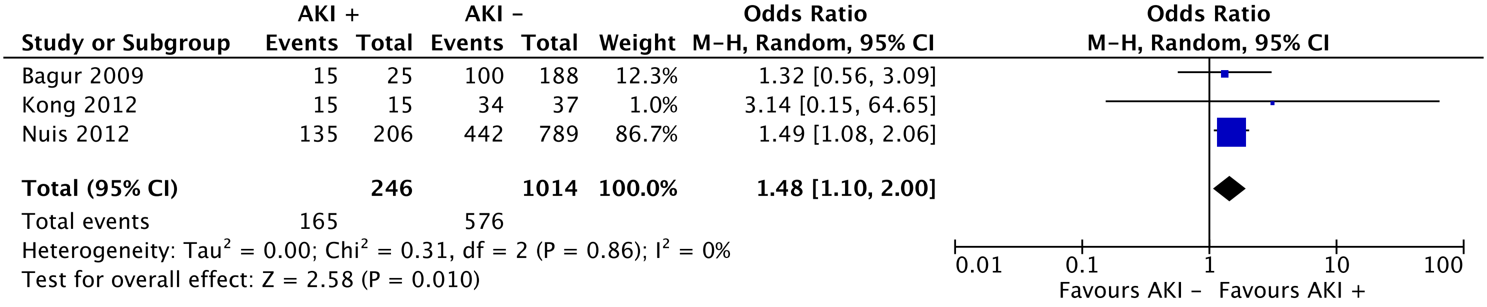

Supplemental figure 5: Meta-analysis of diabetes mellitus in predicting post-TAVR AKI

	AKI +		AKI −			Odds Ratio	Odds Ratio
Study or Subgroup	Events	Total	Events	Total	Weight	M−H, Random, 95% CI	M−H, Random, 95% CI
Aregger 2009	5	15	5	39	6.9%	3.40 [0.82, 14.15]	
Bagur 2009	7	25	41	188	12.1%	1.39 [0.55, 3.57]	
Barbash 2012	5	24	48	141	10.7%	0.51 [0.18, 1.45]	
Elhamidi 2011	17	46	50	188	16.8%	1.62 [0.82, 3.20]	
Genereux 2013	7	18	55	200	11.3%	1.68 [0.62, 4.55]	
Kong 2012	6	15	3	37	5.9%	7.56 [1.57, 36.27]	
Nuis 2012	56	206	218	789	24.5%	0.98 [0.69, 1.38]	
Saia 2013	8	42	15	60	11.7%	0.71 [0.27, 1.86]	
**Total (95% CI)**		**391**		**1642**	**100.0%**	**1.30 [0.85, 2.00]**	
Total events	111		435				

Heterogeneity: $Tau^2 = 0.16$; $Chi^2 = 13.60$, df = 7 (P = 0.06); $I^2 = 49\%$

Test for overall effect: Z = 1.22 (P = 0.22)

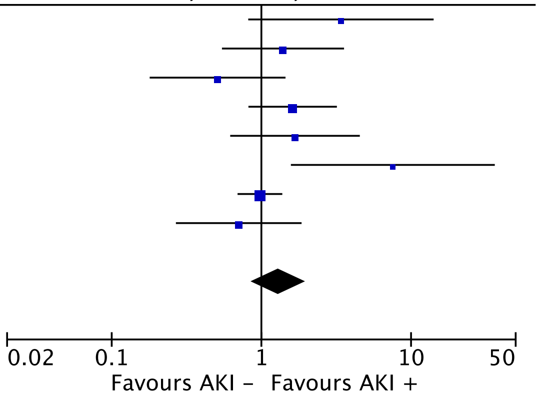

Supplemental figure 6: Meta-analysis of hypertension in predicting post-TAVR AKI

	AKI +		AKI −			Odds Ratio
**Study or Subgroup**	**Events**	**Total**	**Events**	**Total**	**Weight**	**M–H, Random, 95% CI**
Aregger 2009	14	15	31	39	15.5%	3.61 [0.41, 31.72]
Bagur 2009	23	25	127	188	17.2%	5.52 [1.26, 24.19]
Barbash 2012	22	25	133	141	17.3%	0.44 [0.11, 1.79]
Elhamidi 2011	28	24	0	188		Not estimable
Genereux 2013	15	46	0	200	13.7%	197.32 [11.52, 3380.97]
Kong 2012	11	18	11	37	17.8%	3.71 [1.14, 12.10]
Nuis 2012	169	15	169	789		Not estimable
Saia 2013	37	206	37	60	18.6%	0.14 [0.07, 0.26]
**Total (95% CI)**		**374**		**1642**	**100.0%**	**2.54 [0.35, 18.50]**
Total events	319		508			

Heterogeneity: Tau^2 = 5.40; Chi^2 = 65.41, df = 5 (P < 0.00001); I^2 = 92%

Test for overall effect: Z = 0.92 (P = 0.36)

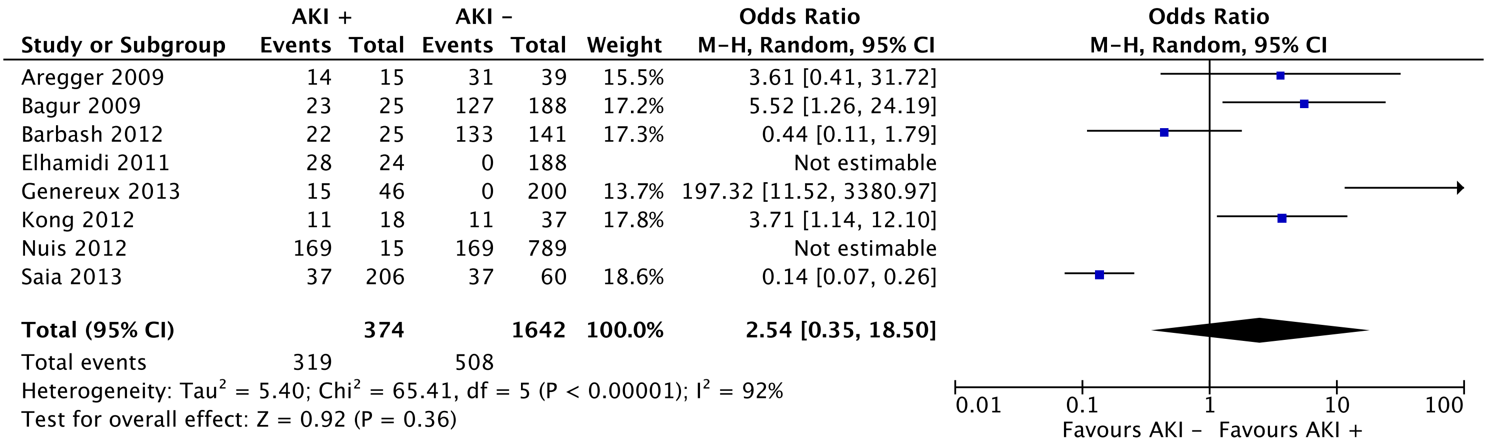

Supplemental figure 7: Meta-analysis of male gender in predicting post-TAVR AKI

	AKI +		AKI −			Odds Ratio
**Study or Subgroup**	**Events**	**Total**	**Events**	**Total**	**Weight**	**M–H, Random, 95% CI**
Aregger 2009	7	15	16	39	3.6%	1.26 [0.38, 4.17]
Bagur 2009	13	25	86	188	7.4%	1.28 [0.56, 2.96]
Barbash 2012	15	24	54	141	6.5%	2.69 [1.10, 6.56]
Elhamidi 2011	15	46	75	188	11.1%	0.73 [0.37, 1.44]
Genereux 2013	9	18	103	200	5.5%	0.94 [0.36, 2.47]
Kong 2012	9	15	24	37	3.4%	0.81 [0.24, 2.79]
Nuis 2012	104	206	393	789	54.8%	1.03 [0.76, 1.40]
Saia 2013	15	42	25	60	7.8%	0.78 [0.34, 1.75]
**Total (95% CI)**		**391**		**1642**	**100.0%**	**1.04 [0.83, 1.31]**
Total events	187		776			

Heterogeneity: $Tau^2 = 0.00$; $Chi^2 = 6.41$, df = 7 (P = 0.49); $I^2 = 0\%$
Test for overall effect: Z = 0.35 (P = 0.73)

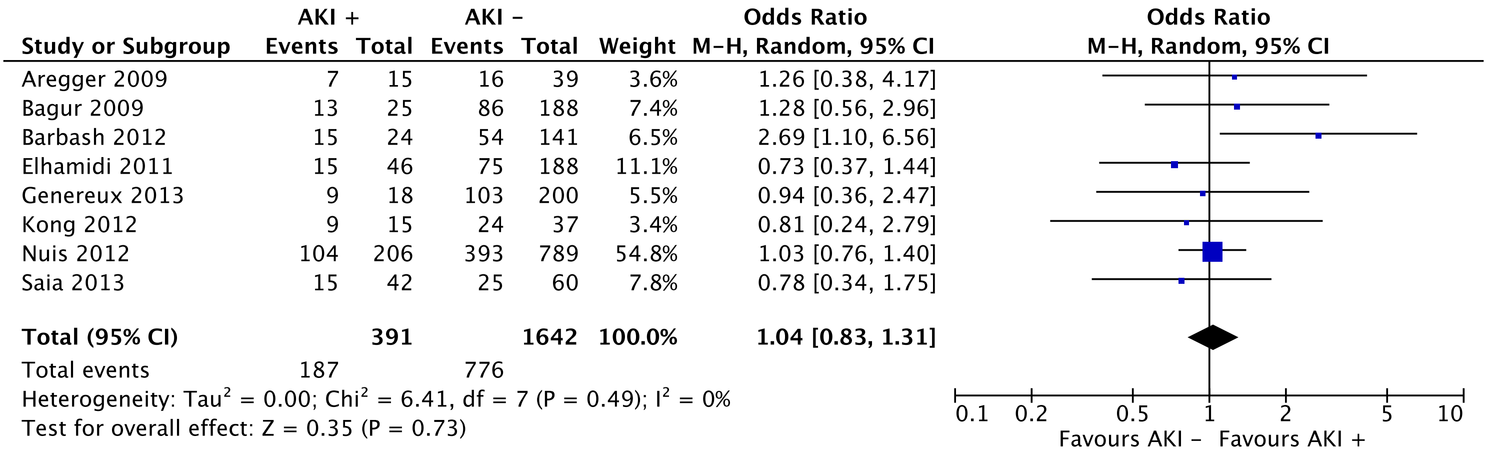

Supplemental figure 8: Meta-analysis of peripheral vascular disease in predicting post-TAVR AKI

	AKI +		AKI −			Odds Ratio
**Study or Subgroup**	**Events**	**Total**	**Events**	**Total**	**Weight**	**M−H, Random, 95% CI**
Aregger 2009	8	15	11	39	4.0%	2.91 [0.85, 9.96]
Bagur 2009	12	25	72	188	8.6%	1.49 [0.64, 3.44]
Barbash 2012	9	24	36	141	7.3%	1.75 [0.71, 4.34]
Elhamidi 2011	16	46	40	188	12.3%	1.97 [0.98, 3.97]
Genereux 2013	6	18	47	200	5.6%	1.63 [0.58, 4.57]
Nuis 2012	79	206	222	789	58.5%	1.59 [1.15, 2.19]
Saia 2013	8	42	4	60	3.7%	3.29 [0.92, 11.77]
**Total (95% CI)**		**376**		**1605**	**100.0%**	**1.72 [1.35, 2.20]**
Total events	138		432			

Heterogeneity: $Tau^2 = 0.00$; $Chi^2 = 2.21$, df = 6 (P = 0.90); $I^2 = 0\%$

Test for overall effect: Z = 4.34 (P < 0.0001)

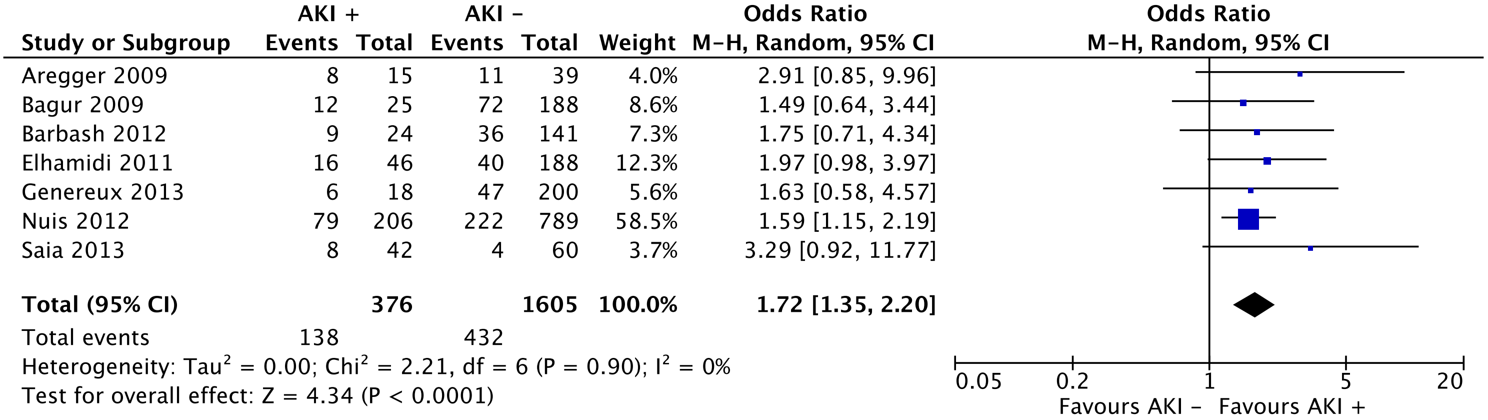

Supplemental figure 9: Meta-analysis of smoking in predicting post-TAVR AKI

	AKI +		AKI −			Odds Ratio
Study or Subgroup	Events	Total	Events	Total	Weight	M-H, Random, 95% CI
Elhamidi 2011	1	46	16	188	43.9%	0.24 [0.03, 1.85]
Genereux 2013	3	18	18	200	56.1%	2.02 [0.53, 7.65]
**Total (95% CI)**		**64**		**388**	**100.0%**	**0.79 [0.09, 7.23]**
Total events	4		34			

Heterogeneity: $Tau^2 = 1.81$; $Chi^2 = 3.33$, df = 1 (P = 0.07); $I^2 = 70\%$
Test for overall effect: Z = 0.21 (P = 0.84)

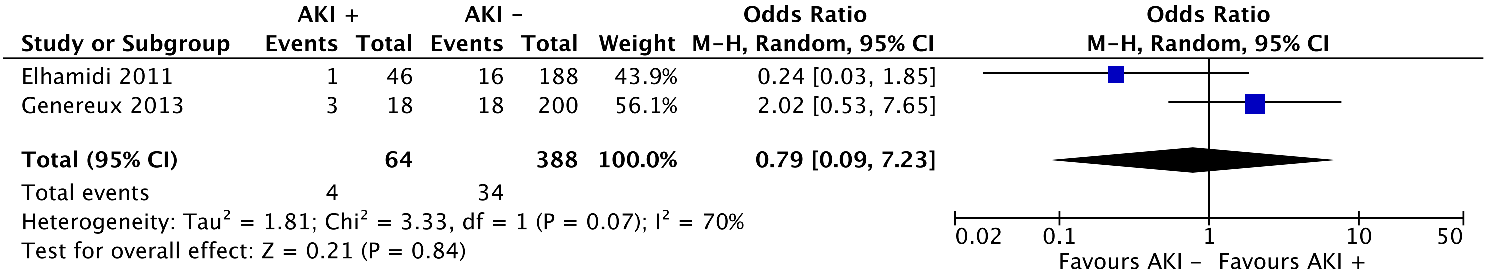

Supplement: Supplementary Materials — Supplemental Figure 1: a meta-analysis of atrial fibrillation in predicting post-TAVR AKI. Supplemental Figure 2: a meta-analysis of blood transfusion in predicting post-TAVR AKI. Supplemental Figure 3: a meta-analysis of coronary artery disease in predicting post-TAVR AKI. Supplemental Figure 4: a meta-analysis of congestive heart failure in predicting post-TAVR AKI. Supplemental Figure 5: a meta-analysis of diabetes mellitus in predicting post-TAVR AKI. Supplemental Figure 6: a meta-analysis of hypertension in predicting post-TAVR AKI. Supplemental Figure 7: a meta-analysis of male gender in predicting post-TAVR AKI. Supplemental Figure 8: a meta-analysis of peripheral vascular disease in predicting post-TAVR AKI. Supplemental Figure 9: a meta-analysis of smoking in predicting post-TAVR AKI. Table 1: risk of bias–comparative observational studies.(). [file 3380605.f1.zip › Supplemental figures.pdf]
