# Supplementary material for: Effect of TAVR Approach and Other Baseline Factors on the Incidence of Acute Kidney Injury: A Systematic Review and Meta-Analysis
Source: J Interv Cardiol. 2022 Oct 27;2022:3380605. doi: 10.1155/2022/3380605 (PMC9633203; doi:10.1155/2022/3380605)
Supplement: Supplementary Materials — Supplemental Figure 1: a meta-analysis of atrial fibrillation in predicting post-TAVR AKI. Supplemental Figure 2: a meta-analysis of blood transfusion in predicting post-TAVR AKI. Supplemental Figure 3: a meta-analysis of coronary artery disease in predicting post-TAVR AKI. Supplemental Figure 4: a meta-analysis of congestive heart failure in predicting post-TAVR AKI. Supplemental Figure 5: a meta-analysis of diabetes mellitus in predicting post-TAVR AKI. Supplemental Figure 6: a meta-analysis of hypertension in predicting post-TAVR AKI. Supplemental Figure 7: a meta-analysis of male gender in predicting post-TAVR AKI. Supplemental Figure 8: a meta-analysis of peripheral vascular disease in predicting post-TAVR AKI. Supplemental Figure 9: a meta-analysis of smoking in predicting post-TAVR AKI. Table 1: risk of bias–comparative observational studies.(). [file 3380605.f1.zip › Supplemental table 1 Study Characteristics.docx]

| **Study** | **Country** | **Study design** | **Year** | **TAVR patients** | | | | **Age** | **Male %** | **Access site** | **Hypertension** | | | | **Diabetes Mellitus** | | | | **Congestive heart failure** | | | | **Coronary artery disease** | | | | **Chronic kidney disease** | | | | **Atrial fibrillation** | | | | **COPD** | | | | **Smoking** | | | | **Peripheral vascular disease** | | | | **Blood transfusion** | | | | **Acute kidney injury** | | | | **AKI timing** | **Follow up** |
| --- | --- | --- | --- | --- | --- | --- | --- | --- | --- | --- | --- | --- | --- | --- | --- | --- | --- | --- | --- | --- | --- | --- | --- | --- | --- | --- | --- | --- | --- | --- | --- | --- | --- | --- | --- | --- | --- | --- | --- | --- | --- | --- | --- | --- | --- | --- | --- | --- | --- | --- | --- | --- | --- | --- | --- | --- |
|  |  |  |  | **Total** | **TF-TAVR** | **TA-TAVR** | **Tao-TAVR** |  |  |  | **Total** | **TF** | **TA** | **Tao** | **Total** | **TF** | **TA** | **Tao** | **Total** | **TF** | **TA** | **Tao** | **Total** | **TF** | **TA** | **Tao** | **Total** | **TF** | **TA** | **Tao** | **Total** | **TF** | **TA** | **Tao** | **Total** | **TF** | **TA** | **Tao** | **Total** | **TF** | **TA** | **Tao** | **Total** | **TF** | **TA** | **Tao** | **Total** | **TF** | **TA** | **Tao** | **Total** | **TF** | **TA** | **Tao** |  |  |
| **Adamo ^1^** | Italy | prospective | 2015 | 322 | 246 | 32 | 44 | 84 ± 7 | 60 | TF, TA, TAo | 231 | 179 | 23 | 29 | 92 | 69 | 8 | 15 | 197 | 155 | 20 | 22 | 147 | 106 | 17 | 24 | NR | NR | NR | NR | 88 | 67 | 15 | 6 | 38 | 30 | 5 | 3 | NR | NR | NR | NR | 87 | 35 | 21 | 31 | NR | NR | NR | NR | 90 | 73 | 4 | 13 | 30 days | 2 years |
| **Agha ^2^** | United States | retrospective | 2019 | 489 | 302 | 187 | na | 81 ± 8.9 | 57 | TF, TA | 459 | 284 | 175 | NR | 173 | 111 | 62 | NR | NR | NR | NR | NR | 378 | 226 | 152 | NR | NR | NR | NR | NR | NR | NR | NR | NR | NR | NR | NR | NR | 265 | 151 | 114 | NR | 150 | 67 | 83 | NR | NR | NR | NR | NR | 26 | 13 | 13 | NR | 30 days | 1 year |
| **Arai ^3^** | France | retrospective | 2016 | 798 | 467 | 42 | 289 | 82.9 | 51.3 | TF, TA, TAo | 536 | 320 | 27 | 189 | 181 | 103 | 18 | 60 | NR | NR | NR | NR | 34 | 15 | 2 | 17 | NR | NR | NR | NR | 210 | 132 | 18 | 60 | 105 | 59 | 8 | 38 | NR | NR | NR | NR | 156 | 49 | 15 | 92 | NR | NR | NR | NR | 72 | 21 | 13 | 38 | 30 days | median 199 days |
| **Aregger ^4^** | Switzerland | retrospective | 2009 | 58 | 46 | 12 | na | 83 ± 5 | 41 | TF, TA | 49 | NR | NR | NR | 12 | NR | NR | NR | NR | NR | NR | NR | 30 | NR | NR | NR | NR | NR | NR | NR | NR | NR | NR | NR | NR | NR | NR | NR | NR | NR | NR | NR | 21 | NR | NR | NR | NR | NR | NR | NR | 15 | 8 | 7 | NR | 15 days | 30 days |
| **Asthana ^5^** | united States | retrospective | 2018 | 84 | 63 | 21 | na | 85.8 | 51 | TF, TA | 147 | 102 | 45 | NR | 57 | 41 | 16 | NR | NR | NR | NR | NR | 67 | 44 | 23 | NR | NR | NR | NR | NR | 65 | 43 | 22 | NR | 61 | 42 | 19 | NR | 7 | 6 | 1 | NR | 17 | 10 | 17 | NR | NR | NR | NR | NR | 39 | 24 | 15 | NR | NR | 30 days |
| **Bagur ^6^** | Canada | prospective | 2009 | 213 | 111 | 102 | na | 82+8 | 47 | TF, TA | 150 | NR | NR | NR | 48 | NR | NR | NR | 115 | NR | NR | NR | 143 | NR | NR | NR | NR | NR | NR | NR | NR | NR | NR | NR | 69 | NR | NR | NR | NR | NR | NR | NR | 84 | NR | NR | NR | 104 | NR | NR | NR | 25 | 9 | 16 | NR | 48 hours | NR |
| **Barbash ^7^** | United States | retrospective | 2011 | 165 | 117 | 48 | na | 84.5 | 41.8 | TF, TA | 158 | NR | NR | NR | 48 | NR | NR | NR | NR | NR | NR | NR | 92 | NR | NR | NR | 128 | NR | NR | NR | NR | NR | NR | NR | 45 | NR | NR | NR | NR | NR | NR | NR | 45 | NR | NR | NR | 70 | NR | NR | NR | 24 | 12 | 12 | NR | 48 hours | 30 days |
| **Biancari ^8^** | European | retrospective | 2016 | 398 | 199 | 199 | na | 81.4 | 55.8 | TF, TA | NR | NR | NR | NR | 102 | 52 | 50 | NR | NR | NR | NR | NR | 104 | 51 | 53 | NR | 10 | 5 | 5 | NR | NR | NR | NR | NR | NR | NR | NR | NR | 76 | 37 | 39 | NR | NR | NR | NR | NR | 156 | 59 | 97 | NR | 124 | 41 | 83 | NR | NR | 3 years |
| **Bona ^9^** | Italy | prospective | 2015 | 264 | 179 | 85 | na | 82 | 35 | Tf, TA | 219 | 147 | 72 | NR | 55 | 35 | 20 | NR | 14 | 7 | 7 | NR | 132 | 83 | 49 | NR | 32 | 19 | 13 | NR | NR | NR | NR | NR | 62 | 42 | 20 | NR | NR | NR | NR | NR | 94 | 53 | 41 | NR | NR | NR | NR | NR | 34 | 15 | 19 | NR | 72 hours | 1 year |
| **Elbadawi ^10^** | United States | retrospective | 2019 | 14543 | 11769 | 2774 | na | 78.8 | 55 | TF, TA | 12201 | 9865 | 2336 | NR | 12201 | 9865 | 2336 | NR | 1605 | 1430 | 175 | NR | 2276 | 1872 | 404 | NR | 5155 | 5037 | 118 | NR | NR | NR | NR | NR | 5227 | 4183 | 1044 | NR | 4016 | 3214 | 802 | NR | 4391 | 3300 | 1091 | NR | 1431 | 581 | 850 | NR | 1203 | 483 | 720 | NR | NR | NR |
| **Elhamidi ^11^** | Germany | prospective | 2011 | 225 | 167 | 54 | 4 | 81 | 39 | TF, TA, TAo | 161 | NR | NR | NR | 67 | NR | NR | NR | 41 | NR | NR | NR | 124 | NR | NR | NR | NR | NR | NR | NR | NR | NR | NR | NR | 48 | NR | NR | NR | 16 | NR | NR | NR | 56 | NR | NR | NR | 165 | NR | NR | NR | 46 | 29 | 11 | 6 | 7 days | 6 month |
| **Escarega ^12^** | United States | retrospective | 2015 | 511 | 396 | 115 | na | 83 ± 2 | 50 | TF, TA | 457 | 351 | 106 | NR | 163 | 131 | 32 | NR | 114 | 83 | 31 | NR | 93 | 74 | 19 | NR | 252 | 195 | 57 | NR | 205 | 159 | 46 | NR | 154 | 115 | 39 | NR | NR | NR | NR | NR | 168 | 106 | 62 | NR | NR | NR | NR | NR | 88 | 52 | 36 | NR | 7 days | 1 year |
| **Ferrari ^13^** | Switzerland | retrospective | 2017 | 180 | 90 | 90 | na | 82 ± 8.6 | 46 | TF, TA | 117 | 61 | 56 | NR | 30 | 12 | 18 | NR | NR | NR | NR | NR | 90 | 36 | 54 | NR | 75 | 37 | 38 | NR | NR | NR | NR | NR | 38 | 9 | 29 | NR | NR | NR | NR | NR | 91 | 20 | 71 | NR | NR | NR | NR | NR | 4 | 1 | 3 | NR | 7 days | 30 days |
| **Gauthier^14^** | Belgium | retrospective | 2015 | 176 | 117 | 59 | na | 85.3 | 51.7 | TF, TA | NR | NR | NR | NR | 35 | 28 | 7 | NR | NR | NR | NR | NR | 126 | 79 | 47 | NR | NR | NR | NR | NR | 71 | 47 | 24 | NR | 53 | 37 | 16 | NR | NR | NR | NR | NR | NR | NR | NR | NR | 54 | 31 | 23 | NR | 31 | 15 | 16 | NR | 48 hours | 1 year |
| **Genereux ^15^** | United States | prospective | 2013 | 218 | 140 | 78 | na | 85.4 ± 7.7 | 52 | TF, TA | 180 | NR | NR | NR | 62 | NR | NR | NR | NR | NR | NR | NR | 88 | NR | NR | NR | NR | NR | NR | NR | 90 | NR | NR | NR | 64 | NR | NR | NR | 21 | NR | NR | NR | 53 | NR | NR | NR | 61 | NR | NR | NR | 18 | 9 | 9 | NR | 7 days | 1 year |
| **Gutmann ^16^** | Germany | retrospective | 2017 | 44141 | 31490 | 12651 | na | 81 | 45 | TF, TA | 27661 | NR | NR | NR | 14701 | NR | NR | NR | 18382 | NR | NR | NR | 20663 | NR | NR | NR | 2159 | NR | NR | NR | 20271 | NR | NR | NR | 6686 | NR | NR | NR | NR | NR | NR | NR | 5470 | NR | NR | NR | NR | NR | NR | NR | 5334 | 3589 | 1745 | NR | NR | NR |
| **Gutmann ^17^** | Germany | retrospective | 2015 | 163 | 97 | 66 | na | 82.2 | 35.6 | TF, TA | 136 | 80 | 56 | NR | 50 | 31 | 19 | NR | NR | NR | NR | NR | 29 | 17 | 12 | NR | NR | NR | NR | NR | NR | NR | NR | NR | 15 | 8 | 7 | NR | NR | NR | NR | NR | 46 | 29 | 17 | NR | NR | NR | NR | NR | 36 | 23 | 13 | NR | 7 days | NR |
| **Hamm ^18^** | Germany | prospective | 2017 | 219 | 120 | 99 | na | 80.9 ± 4.4 | 40.7 | TF, TA | 205 | 114 | 91 | NR | 75 | 35 | 40 | NR | 65 | 23 | 42 | NR | 58 | 28 | 30 | NR | NR | NR | NR | NR | 61 | 25 | 36 | NR | 17 | 9 | 8 | NR | NR | NR | NR | NR | NR | NR | NR | NR | NR | NR | NR | NR | 42 | 12 | 30 | NR | 7 days | 5 years |
| **Köhler ^19^** | Germany | prospective | 2016 | 259 | 148 | 75 | 36 | 82 ± 6 | 44.8 | TF, TA, TAo | 236 | 132 | 72 | 32 | 81 | 48 | 23 | 11 | NR | NR | NR | NR | 189 | 104 | 60 | 25 | NR | NR | NR | NR | 119 | 69 | 35 | 15 | 41 | 21 | 14 | 6 | NR | NR | NR | NR | 60 | 27 | 28 | 5 | NR | NR | NR | NR | 15 | 7 | 5 | 3 | 7 day | 1 year |
| **Koifman ^20^** | United States | prospective | 2016 | 648 | 516 | 132 | na | 83 ± 8 | 50 | TF, TA | 584 | 462 | 122 | NR | 209 | 171 | 38 | NR | 148 | 111 | 37 | NR | 381 | 300 | 81 | NR | NR | NR | NR | NR | 264 | 212 | 52 | NR | 211 | 164 | 47 | NR | NR | NR | NR | NR | 213 | 143 | 70 | NR | 194 | 129 | 65 | NR | 15 | 6 | 9 | NR | 7 days | 1 year |
| **Kong ^21^** | Australia | retrospective | 2012 | 52 | 41 | 11 | na | 84 ± 6 | 63.5 | TF, TA | 29 | NR | NR | NR | 9 | NR | NR | NR | 12 | NR | NR | NR | 40 | NR | NR | NR | 21 | NR | NR | NR | NR | NR | NR | NR | 26 | NR | NR | NR | NR | NR | NR | NR | NR | NR | NR | NR | NR | NR | NR | NR | 15 | 9 | 6 | NR | 48 hours | 1 year |
| **Kowalski ^22^** | Germany | prospective | 2017 | 1644 | 504 | 1140 | na | 81.0 ± 5.8 | 45.7 | TF, TA | 1551 | 462 | 1089 | NR | 502 | 139 | 363 | NR | NR | NR | NR | NR | 937 | 213 | 724 | NR | 935 | 260 | 675 | NR | 664 | 171 | 493 | NR | 332 | 104 | 228 | NR | NR | NR | NR | NR | NR | NR | NR | NR | NR | NR | NR | NR | 10 | 0 | 9 | NR | 7 days | 5 years |
| **Krau ^23^** | Germany | prospective | 2015 | 217 | 123 | 72 | 22 | 81.8 ± 6.0 | 44.2 | TF, TA, TAo | 197 | 110 | 68 | 19 | 64 | 39 | 19 | 6 | 25 | 15 | 7 | 3 | 197 | 110 | 68 | 19 | NR | NR | NR | NR | 97 | 55 | 34 | 8 | 39 | 20 | 15 | 4 | NR | NR | NR | NR | 55 | 25 | 27 | 3 | NR | NR | NR | NR | 11 | 6 | 4 | 1 | 7 days | median 349 days |
| **Murarka^24^** | United States | retrospective | 2015 | 123 | 66 | 57 | na | 82.1 ± 7.7 | 40.3 | TF, TA | 119 | 63 | 56 | NR | 35 | 22 | 13 | NR | NR | NR | NR | NR | 82 | 42 | 40 | NR | 19 | 13 | 6 | NR | NR | NR | NR | NR | 25 | 12 | 13 | NR | NR | NR | NR | NR | NR | NR | NR | NR | NR | NR | NR | NR | 23 | 10 | 13 | NR | 7 days | 1 month |
| **Nuis ^25^** | Multi | prospective | 2016 | 971 | 691 | 277 | 3 | 82 | 50 | TF, TA, TAo | 772 | NR | NR | NR | 274 | NR | NR | NR | 577 | NR | NR | NR | 303 | NR | NR | NR | 735 | NR | NR | NR | 265 | NR | NR | NR | 281 | NR | NR | NR | NR | NR | NR | NR | 301 | NR | NR | NR | 157 | NR | NR | NR | 200 | 130 | 67 | 3 | 72 hours | 1 year |
| **D’Onofrio ^26^** | Italy | retrospective | 2015 | 338 | 233 | 105 | na | 80.3 ± 6.7 | 53.6 | TF, TA | 306 | 209 | 97 | NR | 91 | 62 | 29 | NR | NR | NR | NR | NR | 193 | 129 | 64 | NR | 175 | 116 | 59 | NR | NR | NR | NR | NR | 98 | 71 | 27 | NR | 92 | 61 | 31 | NR | 33 | 13 | 20 | NR | NR | NR | NR | NR | 58 | 26 | 32 | NR | 7 days | 1 month |
| **D’Onofrio ^27^** | Italy | retrospective | 2017 | 208 | 69 | 139 | na | 80.6 ± 6.8 | 57.7 | TF, TA | 170 | 111 | 59 | NR | 81 | 53 | 28 | NR | 208 | 139 | 69 | NR | 103 | 59 | 44 | NR | NR | NR | NR | NR | 58 | 40 | 18 | NR | 51 | 38 | 13 | NR | NR | NR | NR | NR | 74 | 33 | 41 | NR | NR | NR | NR | NR | 17 | 10 | 7 | NR | 7 days | 3 years |
| **Reents ^28^** | Germany | prospective | 2018 | 1130 | 619 | 511 | na | 81 | 44.8 | TF, TA | NR | NR | NR | NR | 382 | 205 | 177 | NR | 1130 | 619 | 511 | NR | 310 | 149 | 161 | NR | NR | NR | NR | NR | 296 | 157 | 139 | NR | 134 | 80 | 54 | NR | NR | NR | NR | NR | 179 | 48 | 131 | NR | 333 | 108 | 225 | NR | 53 | 16 | 37 | NR | 7 days | 1 year |
| **Rougé ^29^** | France | retrospective | 2015 | 150 | 78 | 72 | na | 82.6 ± 6.9 | 45.3 | TF, TA | 89 | 47 | 42 | NR | 42 | 24 | 18 | NR | 39 | 15 | 24 | NR | 70 | 32 | 38 | NR | NR | NR | NR | NR | NR | NR | NR | NR | 24 | 8 | 16 | NR | 46 | 17 | 29 | NR | 25 | 11 | 14 | NR | 42 | 15 | 27 | NR | 16 | 7 | 9 | NR | 7 days | 2 years |
| **Saia ^30^** | Italy | prospective | 2012 | 90 | 66 | 24 | na | 83.7 ± 5.3 | 39.2 | TF, TA | 82 | NR | NR | NR | 23 | NR | NR | NR | 102 | NR | NR | NR | 51 | NR | NR | NR | 89 | NR | NR | NR | NR | NR | NR | NR | 26 | NR | NR | NR | NR | NR | NR | NR | 12 | NR | NR | NR | NR | NR | NR | NR | 36 | 20 | 16 | NR | 72 hours | 1 year |
| **Sawa ^31^** | Japan | prospective | 2014 | 64 | 37 | 27 | na | 84.3 ± 6.1 | 34.4 | TF, TA | NR | NR | NR | NR | NR | NR | NR | NR | 64 | 37 | 27 | NR | 18 | 10 | 8 | NR | NR | NR | NR | NR | NR | NR | NR | NR | 15 | 10 | 5 | NR | NR | NR | NR | NR | NR | NR | NR | NR | NR | NR | NR | NR | 1 | 0 | 1 | NR | 7 days | 6 months |
| **Seiffert ^32^** | Germany | retrospective | 2012 | 326 | 149 | 177 | na | 80.6 (79.8–81.3) | 44.5 | TF, TA | NR | NR | NR | NR | NR | NR | NR | NR | 319 | 144 | 175 | NR | 201 | 79 | 122 | NR | NR | NR | NR | NR | 106 | 34 | 72 | NR | NR | NR | NR | NR | NR | NR | NR | NR | NR | NR | NR | NR | NR | NR | NR | NR | 96 | 34 | 62 | NR | 72 hours | median 257.5 days |
| **Tanawuttiwat ^33^** | United States | retrospective | 2014 | 88 | 28 | 36 | 24 | 85.4 | 45.5 | All 3 | 79 | 24 | 31 | 24 | 31 | 14 | 10 | 7 | 72 | 19 | 31 | 22 | 65 | 18 | 33 | 14 | 37 | 10 | 14 | 13 | NR | NR | NR | NR | 33 | 5 | 15 | 13 | NR | NR | NR | NR | 39 | 7 | 15 | 17 | 72 | 15 | 35 | 22 | 30 | 6 | 16 | 8 | NR | 30 days |
| **Thongprayoona ^34^** | United States | retrospective | 2016 | 366 | 195 | 171 | na | 81 | 56.5 | TF, TA | 331 | 174 | 157 | NR | 147 | 77 | 70 | NR | 208 | 108 | 100 | NR | 132 | 63 | 69 | NR | NR | NR | NR | NR | NR | NR | NR | NR | NR | NR | NR | NR | NR | NR | NR | NR | 211 | 86 | 125 | NR | 120 | 51 | 69 | NR | 101 | 36 | 65 | NR | 7 days | 6 months |
| **Van der Boon ^35^** | European | retrospective | 2014 | 882 | 793 | 89 | na | 81.2 ± 7.0 | 53.3 | TF, TA | 307 | 236 | 71 | NR | 250 | 223 | 27 | NR | 719 | 646 | 73 | NR | 400 | 345 | 55 | NR | NR | NR | NR | NR | NR | NR | NR | NR | 290 | 257 | 33 | NR | NR | NR | NR | NR | 200 | 140 | 60 | NR | NR | NR | NR | NR | 205 | 171 | 34 | NR | 72 | median 365 days |
| **Varela-Lema ^36^** | Spain | prospective | 2014 | 94 | 61 | 28 | na | 82.3 ± 5.2 | 46.8 | TF, TA | 76 | 51 | 20 | NR | 24 | 18 | 5 | NR | 93 | 60 | 28 | NR | NR | NR | NR | NR | 26 | 16 | 8 | NR | NR | NR | NR | NR | 22 | 17 | 4 | NR | NR | NR | NR | NR | NR | NR | NR | NR | NR | NR | NR | NR | 6 | 5 | 1 | NR | NR | 1 year |

**References:**

1. Adamo, M., Fiorina, C., Curello, S., Maffeo, D., Chizzola, G., Di Matteo, G., Mastropierro, R., Nardi, M., Cervi, E., De Cicco, G., Chiari, E., Curnis, A., Bonardelli, S., Coletti, G., Manzato, A., Metra, M., & Ettori, F. (2015). Role of different vascular approaches on transcatheter aortic valve implantation outcome: a single-center study. Journal of cardiovascular medicine (Hagerstown, Md.), 16(4), 279–285. <https://doi.org/10.2459/JCM.0000000000000252>
2. Agha, A. M., Burt, J. R., Beetler, D., Tran, T., Parente, R., Sensakovic, W., Du, Y., & Siddiqui, U. (2019). The Association between Transcatheter Aortic Valve Replacement (TAVR) Approach and New-Onset Bundle Branch Blocks. Cardiology and therapy, 8(2), 357–364. Advance online publication. <https://doi.org/10.1007/s40119-019-0137-2>
3. Arai, T., Romano, M., Lefèvre, T., Hovasse, T., Farge, A., Le Houerou, D., Hayashida, K., Watanabe, Y., Garot, P., Benamer, H., Unterseeh, T., Bouvier, E., Morice, M. C., & Chevalier, B. (2016). Direct Comparison of Feasibility and Safety of Transfemoral Versus Transaortic Versus Transapical Transcatheter Aortic Valve Replacement. JACC. Cardiovascular interventions, 9(22), 2320–2325. <https://doi.org/10.1016/j.jcin.2016.08.009>
4. Aregger, F., Wenaweser, P., Hellige, G. J., Kadner, A., Carrel, T., Windecker, S., & Frey, F. J. (2009). Risk of acute kidney injury in patients with severe aortic valve stenosis undergoing transcatheter valve replacement. Nephrology, dialysis, transplantation : official publication of the European Dialysis and Transplant Association - European Renal Association, 24(7), 2175–2179. <https://doi.org/10.1093/ndt/gfp036>
5. Asthana, N., Mantha, A., Yang, E. H., Suh, W., Aksoy, O., Shemin, R. J., Vorobiof, G., & Benharash, P. (2018). Myocardial functional changes in transfemoral versus transapical aortic valve replacement. The Journal of surgical research, 221, 304–310. <https://doi.org/10.1016/j.jss.2017.08.036>
6. Bagur, R., Webb, J. G., Nietlispach, F., Dumont, E., De Larochellière, R., Doyle, D., Masson, J. B., Gutiérrez, M. J., Clavel, M. A., Bertrand, O. F., Pibarot, P., & Rodés-Cabau, J. (2010). Acute kidney injury following transcatheter aortic valve implantation: predictive factors, prognostic value, and comparison with surgical aortic valve replacement. European heart journal, 31(7), 865–874. <https://doi.org/10.1093/eurheartj/ehp552>
7. Barbash, I. M., Ben-Dor, I., Dvir, D., Maluenda, G., Xue, Z., Torguson, R., Satler, L. F., Pichard, A. D., & Waksman, R. (2012). Incidence and predictors of acute kidney injury after transcatheter aortic valve replacement. American heart journal, 163(6), 1031–1036. <https://doi.org/10.1016/j.ahj.2012.01.009>
8. Biancari, F., Rosato, S., D'Errigo, P., Ranucci, M., Onorati, F., Barbanti, M., Santini, F., Tamburino, C., Santoro, G., Grossi, C., Covello, R. D., Ventura, M., Fusco, D., Seccareccia, F., & OBSERVANT Research Group (2016). Immediate and Intermediate Outcome After Transapical Versus Transfemoral Transcatheter Aortic Valve Replacement. The American journal of cardiology, 117(2), 245–251. <https://doi.org/10.1016/j.amjcard.2015.10.036>
9. Bona, V., Khawaja, M. Z., Bapat, V., Young, C., Hancock, J., Redwood, S., Fusari, M., & Thomas, M. (2015). Early and late changes in quality of life following transcatheter aortic valve implantation using the transfemoral and transapical approaches. EuroIntervention : journal of EuroPCR in collaboration with the Working Group on Interventional Cardiology of the European Society of Cardiology, 11(2), 221–229. <https://doi.org/10.4244/EIJV11I2A41>
10. Elbadawi, A., Mohamed, A. H., Elgendy, I. Y., Ogunbayo, G. O., Megaly, M., Shahin, H. I., Mahmoud, K., Omer, M. A., Abuzaid, A., Fujise, K., & Gilani, S. (2020). Comparative Outcomes of Transapical Versus Transfemoral Access for Transcatheter Aortic Valve Replacement in Diabetics. Cardiology and therapy, 9(1), 107–118. <https://doi.org/10.1007/s40119-019-00155-5>
11. Elhmidi, Y., Bleiziffer, S., Piazza, N., Hutter, A., Opitz, A., Hettich, I., Kornek, M., Ruge, H., Brockmann, G., Mazzitelli, D., & Lange, R. (2011). Incidence and predictors of acute kidney injury in patients undergoing transcatheter aortic valve implantation. American heart journal, 161(4), 735–739. <https://doi.org/10.1016/j.ahj.2011.01.009>
12. Escárcega, R. O., Lipinski, M. J., Baker, N. C., Magalhaes, M. A., Minha, S., Torguson, R., Chen, F., Ben-Dor, I., Satler, L. F., Pichard, A. D., Corso, P., & Waksman, R. (2015). Analysis of long-term survival following transcatheter aortic valve implantation from a single high-volume center. The American journal of cardiology, 116(2), 256–263. <https://doi.org/10.1016/j.amjcard.2015.04.016>
13. Ferrari, E., Eeckhout, E., Keller, S., Muller, O., Tozzi, P., Berdajs, D., & von Segesser, L. K. (2017). Transfemoral versus transapical approach for transcatheter aortic valve implantation: hospital outcome and risk factor analysis. Journal of cardiothoracic surgery, 12(1), 78. <https://doi.org/10.1186/s13019-017-0638-9>
14. Gauthier, C., Astarci, P., Baele, P., Matta, A., Kahn, D., Kefer, J., & Momeni, M. (2015). Mid-term survival after transcatheter aortic valve implantation: Results with respect to the anesthetic management and to the access route (transfemoral versus transapical). Annals of cardiac anaesthesia, 18(3), 343–351. <https://doi.org/10.4103/0971-9784.159804>
15. Généreux, P., Kodali, S. K., Green, P., Paradis, J. M., Daneault, B., Rene, G., Hueter, I., Georges, I., Kirtane, A., Hahn, R. T., Smith, C., Leon, M. B., & Williams, M. R. (2013). Incidence and effect of acute kidney injury after transcatheter aortic valve replacement using the new valve academic research consortium criteria. The American journal of cardiology, 111(1), 100–105. <https://doi.org/10.1016/j.amjcard.2012.08.057>
16. Gutmann, A., Kaier, K., Reinecke, H., Frankenstein, L., Zirlik, A., Bothe, W., von Zur Mühlen, C., Zehender, M., Reinöhl, J., Bode, C., & Stachon, P. (2017). Impact of pulmonary hypertension on in-hospital outcome after surgical or transcatheter aortic valve replacement. EuroIntervention : journal of EuroPCR in collaboration with the Working Group on Interventional Cardiology of the European Society of Cardiology, 13(7), 804–810. <https://doi.org/10.4244/EIJ-D-16-00927>
17. Gutmann, A., Kaier, K., Sorg, S., von Zur Mühlen, C., Siepe, M., Moser, M., Geibel, A., Zirlik, A., Ahrens, I., Baumbach, H., Beyersdorf, F., Vach, W., Zehender, M., Bode, C., & Reinöhl, J. (2015). Analysis of the additional costs of clinical complications in patients undergoing transcatheter aortic valve replacement in the German Health Care System. International journal of cardiology, 179, 231–237. <https://doi.org/10.1016/j.ijcard.2014.11.095>
18. Hamm, K., Reents, W., Zacher, M., Kerber, S., Diegeler, A., Schieffer, B., & Barth, S. (2017). Transcatheter aortic valve implantation using the ACURATE TA and ACURATE neo valves: a four-year single-centre experience. EuroIntervention : journal of EuroPCR in collaboration with the Working Group on Interventional Cardiology of the European Society of Cardiology, 13(1), 53–59. <https://doi.org/10.4244/EIJ-D-16-00898>
19. Köhler, W. M., Freitag-Wolf, S., Lambers, M., Lutz, M., Niemann, P. M., Petzina, R., Lutter, G., Bramlage, P., Frey, N., & Frank, D. (2016). Preprocedural but not periprocedural high-sensitive Troponin T levels predict outcome in patients undergoing transcatheter aortic valve implantation. Cardiovascular therapeutics, 34(6), 385–396. <https://doi.org/10.1111/1755-5922.12208>
20. Koifman, E., Magalhaes, M., Kiramijyan, S., Escarcega, R. O., Didier, R., Torguson, R., Ben-Dor, I., Corso, P., Shults, C., Satler, L., Pichard, A., & Waksman, R. (2016). Impact of transfemoral versus transapical access on mortality among patients with severe aortic stenosis undergoing transcatheter aortic valve replacement. Cardiovascular revascularization medicine : including molecular interventions, 17(5), 318–321. <https://doi.org/10.1016/j.carrev.2016.05.002>
21. Kong, W. Y., Yong, G., & Irish, A. (2012). Incidence, risk factors and prognosis of acute kidney injury after transcatheter aortic valve implantation. Nephrology (Carlton, Vic.), 17(5), 445–451. <https://doi.org/10.1111/j.1440-1797.2012.01593.x>
22. Kowalski, M., Deutsch, C., Hofmann, S., Franz, N., Billion, M., Ferdosi, A., Bramlage, P., Imnadze, G., & Warnecke, H. (2017). Transcatheter aortic valve implantation at a high-volume center: the Bad Rothenfelde experience. Kardiochirurgia i torakochirurgia polska = Polish journal of cardio-thoracic surgery, 14(4), 215–224. <https://doi.org/10.5114/kitp.2017.72224>
23. Krau, N. C., Lünstedt, N. S., Freitag-Wolf, S., Brehm, D., Petzina, R., Lutter, G., Bramlage, P., Dempfle, A., Frey, N., & Frank, D. (2015). Elevated growth differentiation factor 15 levels predict outcome in patients undergoing transcatheter aortic valve implantation. European journal of heart failure, 17(9), 945–955. <https://doi.org/10.1002/ejhf.318>
24. Murarka, S., Lazkani, M., Neihaus, M., Boggess, M., Morris, M., Gellert, G., Fang, H. K., & Pershad, A. (2015). Comparison of 30-Day Outcomes of Transfemoral Versus Transapical Approach for Transcatheter Aortic Valve Replacement: A Single-Center US Experience. The Annals of thoracic surgery, 99(5), 1539–1544. <https://doi.org/10.1016/j.athoracsur.2014.12.041>
25. Nuis, R. J., Rodés-Cabau, J., Sinning, J. M., van Garsse, L., Kefer, J., Bosmans, J., Dager, A. E., van Mieghem, N., Urena, M., Nickenig, G., Werner, N., Maessen, J., Astarci, P., Perez, S., Benitez, L. M., Dumont, E., van Domburg, R. T., & de Jaegere, P. P. (2012). Blood transfusion and the risk of acute kidney injury after transcatheter aortic valve implantation. Circulation. Cardiovascular interventions, 5(5), 680–688. <https://doi.org/10.1161/CIRCINTERVENTIONS.112.971291>
26. D'Onofrio, A., Facchin, M., Besola, L., Manzan, E., Tessari, C., Bizzotto, E., Bianco, R., Tarantini, G., Napodano, M., Fraccaro, C., Buja, P., Covolo, E., Yzeiraj, E., Pittarello, D., Isabella, G., Iliceto, S., & Gerosa, G. (2016). Intermediate Clinical and Hemodynamic Outcomes After Transcatheter Aortic Valve Implantation. The Annals of thoracic surgery, 101(3), 881–888. <https://doi.org/10.1016/j.athoracsur.2015.08.032>
27. D'Onofrio, A., Salizzoni, S., Filippini, C., Agrifoglio, M., Alfieri, O., Chieffo, A., Tarantini, G., Gabbieri, D., Savini, C., Immè, S., Ribichini, F., Cugola, D., Raviola, E., Loi, B., Pompei, E., Cappai, A., Cassese, M., Luzi, G., Aiello, M., Santini, F., … Gerosa, G. (2017). Transapical aortic valve replacement is a safe option in patients with poor left ventricular ejection fraction: results from the Italian Transcatheter Balloon-Expandable Registry (ITER). European journal of cardio-thoracic surgery : official journal of the European Association for Cardio-thoracic Surgery, 52(5), 874–880. <https://doi.org/10.1093/ejcts/ezx227>
28. Reents, W., Barth, S., Griese, D. P., Winkler, S., Babin-Ebell, J., Kerber, S., Diegeler, A., Zacher, M., & Hamm, K. (2019). Transfemoral versus transapical transcatheter aortic valve implantation: a single-centre experience. European journal of cardio-thoracic surgery : official journal of the European Association for Cardio-thoracic Surgery, 55(4), 744–750. <https://doi.org/10.1093/ejcts/ezy363>
29. Rougé, A., Huttin, O., Aslam, R., Vaugrenard, T., Jouve, T., Angioi, M., & Maureira, P. (2015). Mid-term results of 150 TAVI comparing apical versus femoral approaches. Journal of cardiothoracic surgery, 10, 147. <https://doi.org/10.1186/s13019-015-0360-4>
30. Saia, F., Ciuca, C., Taglieri, N., Marrozzini, C., Savini, C., Bordoni, B., Dall'Ara, G., Moretti, C., Pilato, E., Martìn-Suàrez, S., Petridis, F. D., Di Bartolomeo, R., Branzi, A., & Marzocchi, A. (2013). Acute kidney injury following transcatheter aortic valve implantation: incidence, predictors and clinical outcome. International journal of cardiology, 168(2), 1034–1040. <https://doi.org/10.1016/j.ijcard.2012.10.029>
31. Sawa, Y., Takayama, M., Mitsudo, K., Nanto, S., Takanashi, S., Komiya, T., Kuratani, T., Tobaru, T., & Goto, T. (2015). Clinical efficacy of transcatheter aortic valve replacement for severe aortic stenosis in high-risk patients: the PREVAIL JAPAN trial. Surgery today, 45(1), 34–43. <https://doi.org/10.1007/s00595-014-0855-y>
32. Seiffert, M., Schnabel, R., Conradi, L., Diemert, P., Schirmer, J., Koschyk, D., Linder, M., Kersten, J. F., Grosser, A., Wilde, S., Blankenberg, S., Reichenspurner, H., Baldus, S., & Treede, H. (2013). Predictors and outcomes after transcatheter aortic valve implantation using different approaches according to the valve academic research consortium definitions. Catheterization and cardiovascular interventions : official journal of the Society for Cardiac Angiography & Interventions, 82(4), 640–652. <https://doi.org/10.1002/ccd.24751>
33. Tanawuttiwat, T., O'Neill, B. P., Cohen, M. G., Chinthakanan, O., Heldman, A. W., Martinez, C. A., Alfonso, C. E., Mitrani, R. D., Macon, C. J., Carrillo, R. G., Williams, D. B., O'Neill, W. W., & Myerburg, R. J. (2014). New-onset atrial fibrillation after aortic valve replacement: comparison of transfemoral, transapical, transaortic, and surgical approaches. Journal of the American College of Cardiology, 63(15), 1510–1519. <https://doi.org/10.1016/j.jacc.2013.11.046>
34. Thongprayoon, C., Cheungpasitporn, W., Srivali, N., Harrison, A. M., Kittanamongkolchai, W., Greason, K. L., & Kashani, K. B. (2017). Transapical versus transfemoral approach and risk of acute kidney injury following transcatheter aortic valve replacement: a propensity-adjusted analysis. Renal failure, 39(1), 13–18. <https://doi.org/10.1080/0886022X.2016.1244072>
35. Varela-Lema, L., Queiro-Verdes, T., Baz-Alonso, J. A., Cuenca-Castillo, J. J., Durán-Muñoz, D., Gónzalez-Juanatey, J. R., Herrera Noreña, J. M., Iñiguez-Romo, A., López-Otero, D., Pradas-Montilla, G., Rubio-Álvarez, J., Salgado-Fernández, J., Trillo Nouche, R., Vázquez-González, N., & López-García, M. (2015). Post-introduction observation of transcatheter aortic valve implantation in Galicia (Spain). Journal of evaluation in clinical practice, 21(1), 34–42. <https://doi.org/10.1111/jep.12225>
36. van der Boon, R. M., Marcheix, B., Tchetche, D., Chieffo, A., Van Mieghem, N. M., Dumonteil, N., Vahdat, O., Maisano, F., Serruys, P. W., Kappetein, A. P., Fajadet, J., Colombo, A., Carrié, D., van Domburg, R. T., & de Jaegere, P. P. (2014). Transapical versus transfemoral aortic valve implantation: a multicenter collaborative study. The Annals of thoracic surgery, 97(1), 22–28. <https://doi.org/10.1016/j.athoracsur.2013.09.088>
