# Supplementary material for: Effect of TAVR Approach and Other Baseline Factors on the Incidence of Acute Kidney Injury: A Systematic Review and Meta-Analysis
Source: J Interv Cardiol. 2022 Oct 27;2022:3380605. doi: 10.1155/2022/3380605 (PMC9633203; doi:10.1155/2022/3380605)
Supplement: Supplementary Materials — Supplemental Figure 1: a meta-analysis of atrial fibrillation in predicting post-TAVR AKI. Supplemental Figure 2: a meta-analysis of blood transfusion in predicting post-TAVR AKI. Supplemental Figure 3: a meta-analysis of coronary artery disease in predicting post-TAVR AKI. Supplemental Figure 4: a meta-analysis of congestive heart failure in predicting post-TAVR AKI. Supplemental Figure 5: a meta-analysis of diabetes mellitus in predicting post-TAVR AKI. Supplemental Figure 6: a meta-analysis of hypertension in predicting post-TAVR AKI. Supplemental Figure 7: a meta-analysis of male gender in predicting post-TAVR AKI. Supplemental Figure 8: a meta-analysis of peripheral vascular disease in predicting post-TAVR AKI. Supplemental Figure 9: a meta-analysis of smoking in predicting post-TAVR AKI. Table 1: risk of bias–comparative observational studies.(). [file 3380605.f1.zip › Supplemental Table 2 Risk of bias.docx]

Table 1: Risk of bias – Comparative observational studies

| Author, year | Selection | | | | Comparability | Outcome | | | Overall |
| --- | --- | --- | --- | --- | --- | --- | --- | --- | --- |
|  | Representativeness of the cohort 1 | Representativeness of cohort 2 | Ascertainment of exposure | Is it clear that the binary outcome of interest was not present at start of study |  | Ascertainment of outcome: Independent blind assessment | Follow-up long enough for outcomes to occur | Adequacy of follow-up |  |
| Adamo, 2015 | Low risk | Low risk | Low risk | Yes | High risk | High risk | Low risk | Low risk | Low risk |
| Agha, 2019 | Low risk | Low risk | Low risk | Yes | Low risk | High risk | Low risk | Unclear | Low risk |
| Arai, 2016 | Low risk | Low risk | Low risk | Yes | High risk | Unclear | Low risk | Unclear | Moderate risk |
| Aregger, 2009 | Unclear | Unclear | Low risk | Yes | Unclear | High risk | Unclear | Unclear | High risk |
| Asthana, 2018 | Unclear | Unclear | Low risk | Yes | Low risk | Unclear | Low risk | Unclear | Moderate risk |
| Bagur, 2010 | Unclear | Unclear | Low risk | Yes | Unclear | Unclear | Unclear | Unclear | High risk |
| Barbash, 2012 | Low risk | Low risk | Low risk | Yes | Unclear | Unclear | Low risk | Unclear | Moderate risk |
| Biancari, 2015 | Low risk | Low risk | Low risk | Yes | Low risk | Unclear | Low risk | Low risk | Low risk |
| Bona, 2015 | Low risk | Low risk | Low risk | Yes | High risk | High risk | Low risk | High risk | Moderate risk |
| D’Onofrio, 2015 | Low risk | Low risk | Low risk | Yes | High risk | High risk | Low risk | Unclear | Moderate risk |
| D’Onofrio, 2017 | Unclear | Unclear | Low risk | Yes | High risk | Unclear | Low risk | Unclear | Moderate risk |
| Elbadawi, 2019 | Unclear | Unclear | Low risk | Yes | Low risk | Unclear | Low risk | Unclear | Moderate risk |
| Elhmidi, 2011 | Low risk | Low risk | Low risk | Yes | Unclear | Unclear | Low risk | Unclear | Moderate risk |
| Escárcega, 2015 | Low risk | Low risk | Low risk | Yes | High risk | High risk | Low risk | Low risk | Low risk |
| Ferrari, 2017 | Low risk | Low risk | Low risk | Yes | High risk | Unclear | Low risk | Low risk | Low risk |
| Gauthier, 2015 | Unclear | Unclear | Low risk | Yes | Unclear | Unclear | Low risk | Unclear | Moderate risk |
| Généreux, 2013 | Low risk | Low risk | Low risk | Yes | Low risk | Unclear | Low risk | Low risk | Low risk |
| Gutmann, 2015 | Low risk | Low risk | Low risk | Yes | High risk | Unclear | Unclear | Unclear | Moderate risk |
| Gutmann, 2017 | Unclear | Unclear | Low risk | Yes | Unclear | Unclear | Low risk | Unclear | Moderate risk |
| Hamm, 2017 | Low risk | Low risk | Low risk | Yes | High risk | High risk | Low risk | Low risk | Low risk |
| Köhler, 2016 | Unclear | Unclear | Low risk | Yes | Low risk | High risk | Low risk | Unclear | Moderate risk |
| Koifman, 2016 | Unclear | Unclear | Low risk | Yes | Low risk | Unclear | Low risk | Unclear | Moderate risk |
| Kong, 2012 | Low risk | Low risk | Low risk | Yes | Low risk | Unclear | Low risk | Unclear | Low risk |
| Kowalski, 2017 | Low risk | Low risk | Low risk | Yes | High risk | Unclear | Low risk | High risk | Moderate risk |
| Krau, 2015 | Unclear | Unclear | Low risk | Yes | Low risk | Unclear | Low risk | Low risk | Moderate risk |
| Murarka, 2015 | Low risk | Low risk | Low risk | Yes | Low risk | High risk | Low risk | Low risk | Low risk |
| Nuis, 2012 | Unclear | Unclear | Low risk | Yes | High risk | Unclear | Low risk | Unclear | Moderate risk |
| Reents, 2018 | Low risk | Low risk | Low risk | Yes | High risk | Unclear | Low risk | Unclear | Moderate risk |
| Rougé, 2015 | Low risk | Low risk | Low risk | Yes | Low risk | High risk | Low risk | Low risk | Low risk |
| Saia, 2012 | Low risk | Low risk | Low risk | Yes | High risk | High risk | Low risk | Unclear | Moderate risk |
| Sawa, 2014 | Unclear | Unclear | Low risk | Yes | Unclear | Unclear | Low risk | Unclear | Moderate risk |
| Seiffert, 2013 | Low risk | Low risk | Low risk | Yes | High risk | Unclear | Low risk | Low risk | Low risk |
| Tanawuttiwat, 2014 | Low risk | Low risk | Low risk | Yes | High risk | Unclear | Low risk | Unclear | Moderate risk |
| Thongprayoon, 2016 | Low risk | Low risk | Low risk | Yes | Low risk | Unclear | Low risk | Low risk | Low risk |
| van der Boon, 2014 | Low risk | Low risk | Low risk | Yes | High risk | High risk | Low risk | Low risk | Low risk |
| Varela-Lema, 2014 | Low risk | Low risk | Low risk | Yes | Low risk | Unclear | Low risk | Low risk | Low risk |
